# Crystal structure of tri­aqua­(4-cyano­benzoato-κ^2^ *O*,*O*′)(nicotinamide-κ*N* ^1^)zinc 4-cyano­benzoate

**DOI:** 10.1107/S2056989015009743

**Published:** 2015-05-23

**Authors:** Gülçin Şefiye Aşkın, Hacali Necefoğlu, Gamze Yılmaz Nayir, Raziye Çatak Çelik, Tuncer Hökelek

**Affiliations:** a Department of Physics, Hacettepe University, 06800 Beytepe, Ankara, Turkey; b Department of Chemistry, Kafkas University, 36100 Kars, Turkey; c International Scientific Research Centre, Baku State University, 1148 Baku, Azerbaijan; d Scientific and Technological Application and Research Center, Aksaray University, 68100, Aksaray, Turkey

**Keywords:** crystal structure, zinc, transition metal complexes, benzoic acid nicotinamide

## Abstract

The asymmetric unit of the title salt, [Zn(C_8_H_4_NO_2_)(C_6_H_6_N_2_O)(H_2_O)_3_](C_8_H_4_NO_2_), contains one complex cation and one 4-cyano­benzoate (CNB) counter-anion. The Zn^II^ atom in the cation is coordinated by one 4-cyano­benzoate ligand, one nicotinamide (NA) ligand and three water mol­ecules, the CNB anion thereby coordinating in a bidentate *O*,*O*′-mode through the carboxyl­ate group. The latter, together with one water O atom and the N atom of the NA ligand, form a distorted square-planar arrangement, while the considerably distorted octa­hedral coordination sphere of the Zn^II^ atom is completed by the two O atoms of additional water mol­ecules in the axial positions. The dihedral angles between the planar carboxyl­ate groups and the adjacent benzene rings in the two anions are 10.25 (10) and 5.89 (14)°. Inter­molecular O—H⋯O hydrogen bonds link two of the coordinating water mol­ecules to two free CNB anions. In the crystal, further hydrogen-bonding inter­actions are present, namely N—H⋯O, O—H⋯O and C—H⋯O hydrogen bonds that link the mol­ecular components, enclosing *R*_2_^2^(12), *R*_3_^3^(8) and *R*_3_^3^(9) ring motifs and forming layers parallel to (001). π–π contacts between benzene rings [centroid-to-centroid distances = 3.791 (1) and 3.882 (1) Å] may further stabilize the crystal structure.

## Abstract 2

In the title salt, [Zn(C_8_H_4_NO_2_)(C_6_H_6_N_2_O)(H_2_O)_3_](C_8_H_4_NO_2_), inter­molecular O—H⋯O hydrogen bonds link two of the coordinating water mol­ecules to two free 4-cyano­benzoate anions. N—H⋯O, O—H⋯O and C—H⋯O hydrogen bonds link the mol­ecular components, enclosing 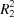
(12), 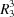
(8) and 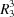
(9) ring motifs and forming layers parallel to (001).

## Chemical context   

As parts of our ongoing investigation on transition-metal complexes of nicotinamide (NA), one form of niacin (Krishnamachari, 1974 ▸), and/or the nicotinic acid derivative *N,N*-di­ethyl­nicotinamide (DENA), an important respiratory stimulant (Bigoli *et al.*, 1972 ▸), the title compound was synthesized and its crystal structure is reported herein.
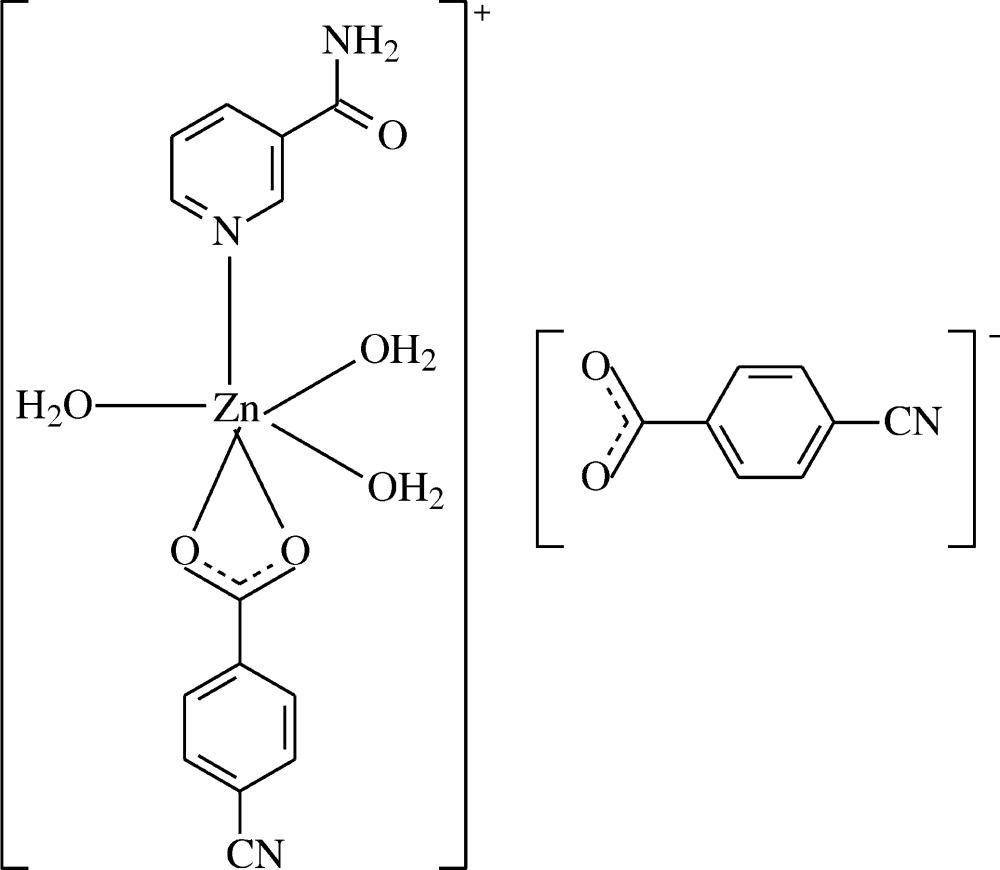


## Structural commentary   

**Figure 1:**
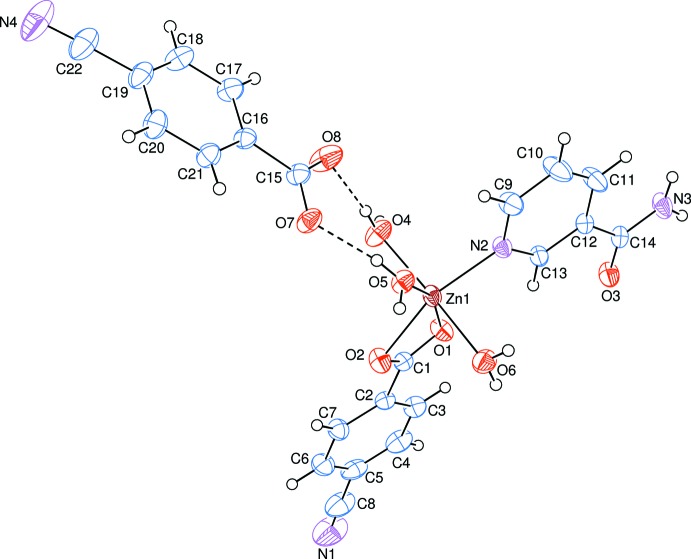
The mol­ecular entities of the title salt, showing the atom-numbering scheme. Displacement ellipsoids are drawn at the 50% probability level. Inter­molecular O—H⋯O hydrogen bonds are shown as dashed lines.

The asymmetric unit of the crystal structure of the title salt, [Zn(C_8_H_4_O_2_N)(C_6_H_6_ON_2_)(H_2_O)_3_](C_8_H_4_O_2_N), is composed of one complex cation and one 4-cyano­benzoate (CNB) counter-anion. The Zn^II^ atom is coordinated by one 4-cyano­benzoate (CNB) anion, one nicotinamide (NA) ligand and three water mol­ecules, the CNB anion and NA ligand coordinating in bidentate and monodentate modes, respectively (Fig. 1 ▸).

In the cation, the four coordinating atoms (O1, O2, O5 and N2) around the Zn1 atom show a distorted square-planar arrangement, while the considerably distorted octa­hedral coordination environment of Zn^II^ is completed by two additional water O atoms (O4 and O6) in the axial positions (Table 1 ▸, Fig. 1 ▸).

The near equality of the C1—O1 [1.2531 (18) Å], C1—O2 [1.2591 (19) Å] and C15—O7 [1.266 (2) Å], C15—O8 [1.237 (2) Å] bonds in the carboxyl­ate groups indicate delocalized bonding arrangements, rather than localized single and double bonds. The average Zn—O bond lengths are 2.19 (11) Å for benzoate oxygen atoms and 2.10 (9) Å for water oxygen atoms; the Zn—N bond length is 2.0545 (12) Å, close to the values in related structures. The Zn1 atom lies 0.0093 (2) Å above the planar (O1/O2/C1) carboxyl­ate group, with a bite angle of 59.48 (4)°. Corresponding O—Zn—O angles are 60.03 (6)° in [Zn(C_9_H_10_NO_2_)(C_6_H_6_N_2_O)·2H_2_O] (Hökelek *et al.*, 2009*a* ▸), 59.02 (8)° in [Zn(C_8_H_8_NO_2_)(C_6_H_6_N_2_O)]·H_2_O (Hökelek *et al.*, 2009*b* ▸) and 57.53 (5), 56.19 (5) and 59.04 (4)° in [Zn(C_8_H_7_O_3_)_2_(C_6_H_6_N_2_O)] (Hökelek *et al.*, 2010 ▸).

The dihedral angles between the planar carboxyl­ate groups [(O1/O2/C1) and (O7/O8/C15)] and the adjacent benzene rings [*A* (C2–C7) and *C* (C16–C21)] are 10.25 (10) and 5.89 (14)°, respectively, while the benzene rings and benzene and pyridine [*B* (N2/C9—13)] rings are oriented at dihedral angles of *A*/*C* = 77.84 (6), *A*/*B* = 8.97 (5) and *B*/*C* = 71.43 (5)°.

## Supra­molecular features   

**Figure 2:**
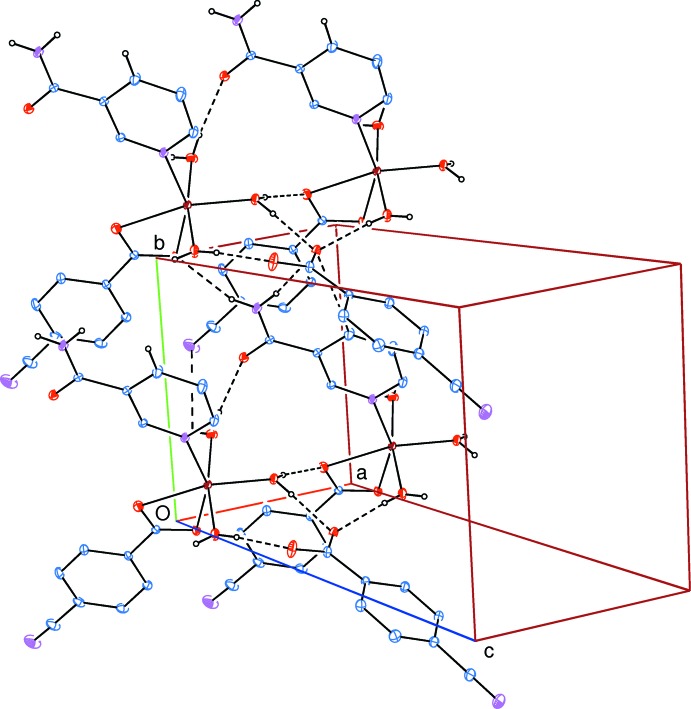
A partial packing diagram of the title complex. Inter­molecular N—H⋯O, O—H⋯O, O—H⋯N and C—H⋯O hydrogen bonds are shown as dashed lines, enclosing 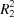
(12), 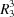
(8) and 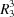
(9) ring motifs. Non-bonding H atoms have been omitted for clarity.

In the crystal, N—H⋯O_c_ (c = carboxyl­ate), O—H_w_⋯O_c_ (w = water), O—H_w_⋯O_n_ (n = nicotinamide), O—H_w_⋯N_n_ as well as C—H_n_⋯O_c_ hydrogen bonds (Table 2 ▸) link the mol­ecular components, enclosing 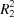
(12), 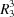
(8) and 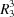
(9) ring motifs (Bernstein *et al.*, 1995 ▸), forming layers parallel to (001) (Fig. 2 ▸). Additional π–π contacts between the benzene rings, *Cg*1⋯*Cg*1^i^ and *Cg*1⋯*Cg*3^ii^ [symmetry codes: (i) 1 − *x*, −*y*, −*z*; (ii) 1 − *x*, −*y*, 1 − *z*, where *Cg*1 and *Cg*3 are the centroids of rings *A* and *C*, respectively], may further stabilize the structure, with centroid-to-centroid distances of 3.791 (1) Å and 3.882 (1) Å, respectively.

## Synthesis and crystallization   

The title compound was prepared by the reaction of ZnSO_4_·7H_2_O (1.44 g, 5 mmol) in H_2_O (30 ml) and nicotinamide (1.22 g, 50 mmol) in H_2_O (50 ml) with sodium 4-cyano­benzoate (1.69 g, 10 mmol) in H_2_O (100 ml). The mixture was filtered and set aside to crystallize at ambient temperature for several days, giving colourless single crystals.

## Refinement   

The experimental details including the crystal data, data collection and refinement are summarized in Table 3 ▸. Atoms H31 and H32 (as part of the NH_2_ group) and H41, H42, H51, H52, H61 and H62 (as part of the water mol­ecules) were located in a difference Fourier map and were refined freely. The aromatic C-bound H atoms were positioned geometrically with C—H = 0.93 Å, and constrained to ride on their parent atoms, with *U*_iso_(H) = 1.2*U*_eq_(C).

## Supplementary Material

Crystal structure: contains datablock(s) I, global. DOI: 10.1107/S2056989015009743/wm5158sup1.cif

Structure factors: contains datablock(s) I. DOI: 10.1107/S2056989015009743/wm5158Isup2.hkl

CCDC reference: 1401948

Additional supporting information:  crystallographic information; 3D view; checkCIF report

## Figures and Tables

**Table 1:** Selected bond lengths ()

Zn1O1	2.2724(12)	Zn1O5	2.0132(11)
Zn1O2	2.1163(12)	Zn1O6	2.1917(14)
Zn1O4	2.0917(13)	Zn1N2	2.0545(12)

**Table 2:** Hydrogen-bond geometry (, )

*D*H*A*	*D*H	H*A*	*D* *A*	*D*H*A*
N3H31O2^i^	0.82(2)	2.13(3)	2.914(2)	162(2)
N3H32O7^i^	0.92(3)	2.35(2)	3.261(2)	171(2)
O4H41O7^ii^	0.75(2)	2.04(2)	2.7890(17)	173(3)
O4H42O8	0.76(3)	1.89(3)	2.6547(18)	175(3)
O5H51O7	0.80(2)	1.83(2)	2.6264(17)	171(3)
O5H52O1^iii^	0.74(2)	2.05(2)	2.7610(17)	164(2)
O6H61O3^iii^	0.75(3)	2.05(3)	2.7993(19)	170(3)
O6H62N1^iv^	0.76(3)	2.17(3)	2.918(3)	170(3)
C11H11O7^i^	0.93	2.49	3.415(2)	177

Symmetry codes: (i) 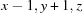; (ii) 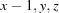; (iii) 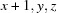; (iv) 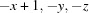.

**Table 3:** Experimental details

Crystal data	
Chemical formula	[Zn(C_8_H_4_NO_2_)(C_6_H_6_N_2_O)(H_2_O)_3_](C_8_H_4_NO_2_)
*M*_r_	533.81
Crystal system, space group	Triclinic, *P* 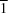
Temperature (K)	296
*a*, *b*, *c* ()	6.0858(2), 8.7031(3), 22.2357(6)
, , ()	81.882(2), 87.806(3), 88.007(3)
*V* (^3^)	1164.55(6)
*Z*	2
Radiation type	Mo *K*
(mm^1^)	1.11
Crystal size (mm)	0.45 0.36 0.25

Data collection	
Diffractometer	Bruker SMART BREEZE CCD
Absorption correction	Multi-scan (*SADABS*; Bruker, 2012 ▸)
*T*_min_, *T*_max_	0.625, 0.758
No. of measured, independent and observed [*I* > 2(*I*)] reflections	27167, 5839, 5450
*R*_int_	0.034
(sin /)_max_ (^1^)	0.670

Refinement	
*R*[*F* ^2^ > 2(*F* ^2^)], *wR*(*F* ^2^), *S*	0.030, 0.080, 1.05
No. of reflections	5839
No. of parameters	348
H-atom treatment	H atoms treated by a mixture of independent and constrained refinement
_max_, _min_ (e ^3^)	0.35, 0.33

Computer programs: *APEX2* and *SAINT* (Bruker, 2012 ▸), *SHELXS97* and *SHELXL97* (Sheldrick, 2008 ▸), *ORTEP-3 for Windows* and *WinGX* (Farrugia, 2012 ▸) and *PLATON* (Spek, 2009 ▸).

## supplementary crystallographic information

### Crystal data

**Table:**

[Zn(C_8_H_4_NO_2_)(C_6_H_6_N_2_O)(H_2_O)_3_](C_8_H_4_NO_2_)	*Z* = 2
*M**_r_* = 533.81	*F*(000) = 548
Triclinic, *P*1	*D*_x_ = 1.522 Mg m^−^^3^
Hall symbol: -P 1	Mo *K*α radiation, λ = 0.71073 Å
*a* = 6.0858 (2) Å	Cell parameters from 9885 reflections
*b* = 8.7031 (3) Å	θ = 2.4–28.4°
*c* = 22.2357 (6) Å	µ = 1.11 mm^−^^1^
α = 81.882 (2)°	*T* = 296 K
β = 87.806 (3)°	Prism, translucent light colourless
γ = 88.007 (3)°	0.45 × 0.36 × 0.25 mm
*V* = 1164.55 (6) Å^3^	

### Data collection

**Table:**

Bruker SMART BREEZE CCD diffractometer	5839 independent reflections
Radiation source: fine-focus sealed tube	5450 reflections with *I* > 2σ(*I*)
Graphite monochromator	*R*_int_ = 0.034
φ and ω scans	θ_max_ = 28.4°, θ_min_ = 1.9°
Absorption correction: multi-scan (*SADABS*; Bruker, 2012)	*h* = −7→8
*T*_min_ = 0.625, *T*_max_ = 0.758	*k* = −11→11
27167 measured reflections	*l* = −29→29

### Refinement

**Table:**

Refinement on *F*^2^	Primary atom site location: structure-invariant direct methods
Least-squares matrix: full	Secondary atom site location: difference Fourier map
*R*[*F*^2^ > 2σ(*F*^2^)] = 0.030	Hydrogen site location: inferred from neighbouring sites
*wR*(*F*^2^) = 0.080	H atoms treated by a mixture of independent and constrained refinement
*S* = 1.05	*w* = 1/[σ^2^(*F*_o_^2^) + (0.0394*P*)^2^ + 0.4376*P*] where *P* = (*F*_o_^2^ + 2*F*_c_^2^)/3
5839 reflections	(Δ/σ)_max_ = 0.001
348 parameters	Δρ_max_ = 0.35 e Å^−^^3^
0 restraints	Δρ_min_ = −0.33 e Å^−^^3^

### Special details

**Table:**

Geometry. All esds (except the esd in the dihedral angle between two l.s. planes) are estimated using the full covariance matrix. The cell esds are taken into account individually in the estimation of esds in distances, angles and torsion angles; correlations between esds in cell parameters are only used when they are defined by crystal symmetry. An approximate (isotropic) treatment of cell esds is used for estimating esds involving l.s. planes.
Refinement. Refinement of F^2^ against ALL reflections. The weighted R-factor wR and goodness of fit S are based on F^2^, conventional R-factors R are based on F, with F set to zero for negative F^2^. The threshold expression of F^2^ > 2sigma(F^2^) is used only for calculating R-factors(gt) etc. and is not relevant to the choice of reflections for refinement. R-factors based on F^2^ are statistically about twice as large as those based on F, and R- factors based on ALL data will be even larger.

### Fractional atomic coordinates and isotropic or equivalent isotropic displacement parameters (Å^2^)

**Table:**

	*x*	*y*	*z*	*U*_iso_*/*U*_eq_	
Zn1	0.90164 (3)	0.228852 (18)	0.220630 (8)	0.02931 (6)	
O1	0.58663 (19)	0.18054 (14)	0.17586 (6)	0.0398 (3)	
O2	0.89148 (19)	0.04418 (14)	0.16835 (6)	0.0386 (3)	
O3	0.1991 (2)	0.64093 (14)	0.17635 (6)	0.0425 (3)	
O4	0.8145 (2)	0.08998 (17)	0.30208 (6)	0.0456 (3)	
H41	0.698 (4)	0.078 (3)	0.3136 (11)	0.051 (7)*	
H42	0.887 (4)	0.079 (3)	0.3298 (12)	0.060 (7)*	
O5	1.21393 (19)	0.21434 (14)	0.24815 (6)	0.0346 (2)	
H51	1.258 (4)	0.162 (3)	0.2779 (11)	0.057 (7)*	
H52	1.298 (4)	0.209 (2)	0.2238 (9)	0.035 (5)*	
O6	1.0470 (3)	0.36922 (16)	0.14029 (6)	0.0442 (3)	
H61	1.087 (4)	0.447 (3)	0.1457 (11)	0.052 (7)*	
H62	0.983 (4)	0.379 (3)	0.1113 (12)	0.060 (8)*	
O7	1.38028 (19)	0.02307 (15)	0.33815 (5)	0.0413 (3)	
O8	1.0769 (2)	0.0341 (2)	0.39584 (6)	0.0620 (4)	
N1	0.1949 (5)	−0.3612 (3)	−0.03004 (10)	0.0940 (9)	
N2	0.7668 (2)	0.42834 (14)	0.24805 (6)	0.0315 (3)	
N3	0.2052 (3)	0.83017 (18)	0.23482 (8)	0.0454 (4)	
H31	0.101 (4)	0.872 (3)	0.2162 (11)	0.058 (7)*	
H32	0.269 (4)	0.879 (3)	0.2633 (11)	0.057 (6)*	
N4	1.7600 (4)	−0.5182 (3)	0.61042 (11)	0.0876 (8)	
C1	0.6926 (2)	0.07134 (17)	0.15590 (7)	0.0292 (3)	
C2	0.5848 (2)	−0.02630 (17)	0.11607 (6)	0.0294 (3)	
C3	0.3787 (3)	0.0185 (2)	0.09399 (8)	0.0381 (3)	
H3	0.3076	0.1079	0.1044	0.046*	
C4	0.2796 (3)	−0.0701 (2)	0.05652 (8)	0.0469 (4)	
H4	0.1420	−0.0405	0.0413	0.056*	
C5	0.3867 (3)	−0.2038 (2)	0.04169 (8)	0.0470 (4)	
C6	0.5917 (4)	−0.2502 (2)	0.06388 (9)	0.0473 (4)	
H6	0.6618	−0.3402	0.0538	0.057*	
C7	0.6911 (3)	−0.16065 (19)	0.10140 (8)	0.0371 (3)	
H7	0.8286	−0.1905	0.1167	0.045*	
C8	0.2809 (5)	−0.2932 (3)	0.00172 (10)	0.0665 (7)	
C9	0.8671 (3)	0.4895 (2)	0.29107 (8)	0.0417 (4)	
H9	0.9926	0.4393	0.3078	0.050*	
C10	0.7913 (3)	0.6238 (3)	0.31145 (10)	0.0558 (5)	
H10	0.8666	0.6654	0.3407	0.067*	
C11	0.6017 (3)	0.6963 (2)	0.28798 (9)	0.0491 (5)	
H11	0.5467	0.7867	0.3017	0.059*	
C12	0.4942 (2)	0.63340 (16)	0.24389 (7)	0.0299 (3)	
C13	0.5853 (3)	0.50013 (16)	0.22481 (7)	0.0303 (3)	
H13	0.5169	0.4585	0.1943	0.036*	
C14	0.2868 (3)	0.70160 (16)	0.21562 (7)	0.0317 (3)	
C15	1.2702 (3)	−0.0089 (2)	0.38754 (7)	0.0346 (3)	
C16	1.3816 (3)	−0.11192 (18)	0.43897 (7)	0.0318 (3)	
C17	1.2673 (3)	−0.1557 (2)	0.49338 (8)	0.0423 (4)	
H17	1.1254	−0.1161	0.4993	0.051*	
C18	1.3630 (3)	−0.2582 (2)	0.53905 (8)	0.0503 (5)	
H18	1.2857	−0.2876	0.5755	0.060*	
C19	1.5746 (3)	−0.3167 (2)	0.53011 (8)	0.0446 (4)	
C20	1.6928 (3)	−0.2701 (2)	0.47640 (9)	0.0445 (4)	
H20	1.8358	−0.3079	0.4708	0.053*	
C21	1.5961 (3)	−0.1669 (2)	0.43142 (8)	0.0379 (3)	
H21	1.6757	−0.1340	0.3957	0.046*	
C22	1.6768 (4)	−0.4285 (3)	0.57586 (10)	0.0600 (6)	

### Atomic displacement parameters (Å^2^)

**Table:**

	*U*^11^	*U*^22^	*U*^33^	*U*^12^	*U*^13^	*U*^23^
Zn1	0.02640 (10)	0.02670 (9)	0.03590 (11)	0.00369 (6)	−0.00771 (7)	−0.00734 (7)
O1	0.0353 (6)	0.0404 (6)	0.0465 (7)	0.0023 (5)	−0.0002 (5)	−0.0171 (5)
O2	0.0304 (6)	0.0382 (6)	0.0498 (7)	0.0022 (5)	−0.0125 (5)	−0.0126 (5)
O3	0.0435 (7)	0.0344 (6)	0.0514 (7)	0.0022 (5)	−0.0162 (6)	−0.0087 (5)
O4	0.0294 (7)	0.0607 (8)	0.0418 (7)	−0.0051 (6)	−0.0061 (6)	0.0118 (6)
O5	0.0247 (5)	0.0420 (6)	0.0354 (6)	0.0037 (5)	−0.0037 (5)	0.0001 (5)
O6	0.0579 (8)	0.0391 (7)	0.0365 (7)	−0.0094 (6)	−0.0098 (6)	−0.0044 (5)
O7	0.0318 (6)	0.0549 (7)	0.0333 (6)	0.0080 (5)	−0.0017 (5)	0.0055 (5)
O8	0.0332 (7)	0.1071 (13)	0.0398 (7)	0.0214 (7)	−0.0021 (5)	0.0051 (7)
N1	0.135 (2)	0.0930 (17)	0.0616 (13)	−0.0520 (16)	−0.0348 (14)	−0.0149 (12)
N2	0.0299 (6)	0.0296 (6)	0.0357 (7)	0.0044 (5)	−0.0043 (5)	−0.0070 (5)
N3	0.0446 (9)	0.0346 (7)	0.0583 (10)	0.0146 (6)	−0.0166 (8)	−0.0113 (7)
N4	0.0892 (16)	0.0843 (15)	0.0815 (15)	−0.0089 (13)	−0.0451 (13)	0.0280 (12)
C1	0.0297 (7)	0.0299 (7)	0.0277 (7)	−0.0022 (5)	−0.0016 (5)	−0.0031 (5)
C2	0.0284 (7)	0.0334 (7)	0.0267 (7)	−0.0051 (6)	−0.0011 (5)	−0.0043 (5)
C3	0.0307 (8)	0.0458 (9)	0.0380 (8)	−0.0017 (7)	−0.0046 (6)	−0.0049 (7)
C4	0.0367 (9)	0.0646 (12)	0.0392 (9)	−0.0136 (8)	−0.0115 (7)	−0.0005 (8)
C5	0.0598 (12)	0.0532 (10)	0.0296 (8)	−0.0268 (9)	−0.0066 (8)	−0.0038 (7)
C6	0.0640 (12)	0.0378 (9)	0.0433 (9)	−0.0094 (8)	−0.0034 (8)	−0.0138 (7)
C7	0.0378 (9)	0.0358 (8)	0.0389 (8)	−0.0015 (6)	−0.0039 (7)	−0.0086 (6)
C8	0.0899 (17)	0.0679 (14)	0.0446 (11)	−0.0359 (13)	−0.0173 (11)	−0.0057 (10)
C9	0.0352 (9)	0.0491 (9)	0.0435 (9)	0.0117 (7)	−0.0118 (7)	−0.0156 (7)
C10	0.0502 (11)	0.0651 (12)	0.0609 (12)	0.0198 (9)	−0.0255 (9)	−0.0379 (10)
C11	0.0477 (10)	0.0479 (10)	0.0580 (11)	0.0169 (8)	−0.0167 (9)	−0.0292 (9)
C12	0.0306 (7)	0.0262 (6)	0.0324 (7)	0.0019 (5)	−0.0020 (6)	−0.0028 (5)
C13	0.0314 (7)	0.0262 (6)	0.0338 (7)	0.0008 (5)	−0.0052 (6)	−0.0056 (5)
C14	0.0323 (8)	0.0248 (6)	0.0369 (8)	0.0010 (5)	−0.0034 (6)	−0.0007 (5)
C15	0.0281 (7)	0.0446 (8)	0.0308 (7)	0.0026 (6)	−0.0058 (6)	−0.0048 (6)
C16	0.0306 (7)	0.0363 (7)	0.0292 (7)	−0.0011 (6)	−0.0064 (6)	−0.0055 (6)
C17	0.0365 (9)	0.0542 (10)	0.0354 (8)	0.0005 (7)	−0.0010 (7)	−0.0041 (7)
C18	0.0545 (11)	0.0608 (12)	0.0331 (9)	−0.0055 (9)	−0.0020 (8)	0.0031 (8)
C19	0.0488 (10)	0.0434 (9)	0.0407 (9)	−0.0062 (8)	−0.0185 (8)	0.0028 (7)
C20	0.0351 (9)	0.0472 (9)	0.0496 (10)	0.0035 (7)	−0.0121 (7)	0.0003 (8)
C21	0.0334 (8)	0.0428 (8)	0.0362 (8)	0.0003 (7)	−0.0040 (6)	−0.0007 (7)
C22	0.0617 (13)	0.0613 (13)	0.0544 (12)	−0.0092 (10)	−0.0247 (10)	0.0090 (10)

### Geometric parameters (Å, º)

**Table:**

Zn1—O1	2.2724 (12)	C5—C4	1.388 (3)
Zn1—O2	2.1163 (12)	C5—C6	1.385 (3)
Zn1—O4	2.0917 (13)	C5—C8	1.444 (3)
Zn1—O5	2.0132 (11)	C6—H6	0.9300
Zn1—O6	2.1917 (14)	C7—C6	1.387 (2)
Zn1—N2	2.0545 (12)	C7—H7	0.9300
Zn1—C1	2.5276 (15)	C9—C10	1.372 (2)
O1—C1	1.2531 (18)	C9—H9	0.9300
O2—C1	1.2591 (19)	C10—H10	0.9300
O3—C14	1.230 (2)	C11—C10	1.380 (3)
O4—H41	0.75 (3)	C11—H11	0.9300
O4—H42	0.77 (3)	C12—C11	1.384 (2)
O5—H51	0.80 (3)	C12—C13	1.382 (2)
O5—H52	0.74 (2)	C12—C14	1.498 (2)
O6—H61	0.75 (3)	C13—H13	0.9300
O6—H62	0.76 (3)	C14—N3	1.327 (2)
O7—C15	1.266 (2)	C15—O8	1.237 (2)
N1—C8	1.136 (3)	C15—C16	1.516 (2)
N2—C9	1.335 (2)	C16—C17	1.384 (2)
N2—C13	1.3351 (19)	C16—C21	1.387 (2)
N3—H31	0.82 (3)	C17—C18	1.385 (3)
N3—H32	0.91 (3)	C17—H17	0.9300
N4—C22	1.136 (3)	C18—H18	0.9300
C1—C2	1.494 (2)	C19—C18	1.386 (3)
C2—C3	1.388 (2)	C19—C22	1.447 (3)
C2—C7	1.390 (2)	C20—C19	1.388 (3)
C3—C4	1.380 (2)	C20—H20	0.9300
C3—H3	0.9300	C21—C20	1.381 (2)
C4—H4	0.9300	C21—H21	0.9300
			
O2—Zn1—O1	59.48 (4)	C5—C4—H4	120.2
O4—Zn1—O1	92.90 (5)	C4—C5—C8	118.5 (2)
O5—Zn1—O1	162.17 (5)	C6—C5—C4	121.15 (16)
O6—Zn1—O1	95.68 (5)	C6—C5—C8	120.4 (2)
N2—Zn1—O1	91.98 (5)	C5—C6—C7	119.12 (18)
O5—Zn1—O2	102.69 (5)	C5—C6—H6	120.4
O4—Zn1—O2	93.77 (6)	C7—C6—H6	120.4
N2—Zn1—O2	150.78 (5)	C2—C7—H7	120.1
O5—Zn1—O4	87.85 (6)	C6—C7—C2	119.86 (17)
N2—Zn1—O4	93.81 (6)	C6—C7—H7	120.1
O2—Zn1—O6	87.82 (5)	N1—C8—C5	178.7 (3)
O4—Zn1—O6	170.83 (6)	N2—C9—C10	122.20 (16)
O5—Zn1—O6	82.99 (6)	N2—C9—H9	118.9
N2—Zn1—O6	89.13 (5)	C10—C9—H9	118.9
O5—Zn1—N2	105.76 (5)	C9—C10—C11	119.10 (17)
O1—Zn1—C1	29.66 (4)	C9—C10—H10	120.5
O2—Zn1—C1	29.82 (5)	C11—C10—H10	120.5
O4—Zn1—C1	93.91 (5)	C10—C11—C12	119.40 (16)
O5—Zn1—C1	132.51 (5)	C10—C11—H11	120.3
O6—Zn1—C1	91.96 (5)	C12—C11—H11	120.3
N2—Zn1—C1	121.41 (5)	C11—C12—C14	123.93 (14)
C1—O1—Zn1	86.53 (9)	C13—C12—C11	117.67 (14)
C1—O2—Zn1	93.49 (9)	C13—C12—C14	118.40 (14)
Zn1—O4—H41	123.9 (18)	N2—C13—C12	123.08 (14)
Zn1—O4—H42	122.6 (19)	N2—C13—H13	118.5
H42—O4—H41	107 (3)	C12—C13—H13	118.5
Zn1—O5—H51	126.1 (17)	O3—C14—N3	122.30 (15)
Zn1—O5—H52	114.8 (16)	O3—C14—C12	121.07 (14)
H52—O5—H51	107 (2)	N3—C14—C12	116.63 (15)
Zn1—O6—H61	115.0 (18)	O7—C15—C16	117.31 (14)
Zn1—O6—H62	118 (2)	O8—C15—O7	124.46 (15)
H61—O6—H62	110 (3)	O8—C15—C16	118.19 (15)
C9—N2—Zn1	118.11 (11)	C17—C16—C15	120.11 (14)
C13—N2—Zn1	123.38 (10)	C17—C16—C21	119.38 (15)
C9—N2—C13	118.51 (13)	C21—C16—C15	120.49 (14)
C14—N3—H31	115.4 (17)	C16—C17—C18	120.40 (17)
C14—N3—H32	123.8 (15)	C16—C17—H17	119.8
H31—N3—H32	120 (2)	C18—C17—H17	119.8
O1—C1—Zn1	63.82 (8)	C17—C18—C19	119.64 (17)
O2—C1—Zn1	56.69 (8)	C17—C18—H18	120.2
C2—C1—Zn1	175.80 (11)	C19—C18—H18	120.2
O1—C1—O2	120.51 (14)	C18—C19—C20	120.37 (16)
O1—C1—C2	120.21 (14)	C18—C19—C22	121.19 (19)
O2—C1—C2	119.28 (13)	C20—C19—C22	118.43 (19)
C3—C2—C1	119.43 (14)	C19—C20—H20	120.3
C3—C2—C7	120.58 (15)	C21—C20—C19	119.37 (17)
C7—C2—C1	119.99 (14)	C21—C20—H20	120.3
C2—C3—H3	120.2	C16—C21—H21	119.6
C4—C3—C2	119.68 (17)	C20—C21—C16	120.76 (16)
C4—C3—H3	120.2	C20—C21—H21	119.6
C3—C4—C5	119.60 (17)	N4—C22—C19	178.0 (3)
C3—C4—H4	120.2		
			
O2—Zn1—O1—C1	−0.14 (9)	C9—N2—C13—C12	−1.1 (2)
O4—Zn1—O1—C1	−92.81 (10)	O1—C1—C2—C3	9.5 (2)
O5—Zn1—O1—C1	−0.8 (2)	O1—C1—C2—C7	−170.49 (14)
O6—Zn1—O1—C1	83.95 (10)	O2—C1—C2—C3	−169.34 (14)
N2—Zn1—O1—C1	173.27 (9)	O2—C1—C2—C7	10.6 (2)
O1—Zn1—O2—C1	0.14 (8)	C1—C2—C3—C4	179.15 (15)
O4—Zn1—O2—C1	91.28 (10)	C7—C2—C3—C4	−0.8 (2)
O5—Zn1—O2—C1	179.92 (9)	C1—C2—C7—C6	−179.31 (15)
O6—Zn1—O2—C1	−97.77 (10)	C3—C2—C7—C6	0.7 (2)
N2—Zn1—O2—C1	−13.46 (16)	C2—C3—C4—C5	0.5 (3)
O1—Zn1—N2—C9	163.71 (13)	C6—C5—C4—C3	0.1 (3)
O1—Zn1—N2—C13	−16.72 (13)	C8—C5—C4—C3	−179.13 (17)
O2—Zn1—N2—C9	175.39 (12)	C4—C5—C6—C7	−0.2 (3)
O2—Zn1—N2—C13	−5.04 (19)	C8—C5—C6—C7	178.95 (17)
O4—Zn1—N2—C9	70.67 (14)	C2—C7—C6—C5	−0.1 (3)
O4—Zn1—N2—C13	−109.76 (13)	N2—C9—C10—C11	1.8 (4)
O5—Zn1—N2—C9	−18.17 (14)	C12—C11—C10—C9	−0.9 (4)
O5—Zn1—N2—C13	161.40 (12)	C13—C12—C11—C10	−0.8 (3)
O6—Zn1—N2—C9	−100.64 (14)	C14—C12—C11—C10	179.55 (19)
O6—Zn1—N2—C13	78.93 (13)	C11—C12—C13—N2	1.9 (2)
C1—Zn1—N2—C9	167.60 (12)	C14—C12—C13—N2	−178.46 (14)
C1—Zn1—N2—C13	−12.83 (15)	C11—C12—C14—O3	179.62 (17)
O1—Zn1—C1—O2	−179.76 (15)	C11—C12—C14—N3	0.1 (3)
O2—Zn1—C1—O1	179.76 (15)	C13—C12—C14—O3	0.0 (2)
O4—Zn1—C1—O1	88.99 (10)	C13—C12—C14—N3	−179.54 (15)
O4—Zn1—C1—O2	−90.77 (10)	O7—C15—C16—C17	−177.65 (16)
O5—Zn1—C1—O1	179.65 (9)	O7—C15—C16—C21	0.4 (2)
O5—Zn1—C1—O2	−0.11 (12)	O8—C15—C16—C17	0.1 (3)
O6—Zn1—C1—O1	−98.06 (10)	O8—C15—C16—C21	178.16 (18)
O6—Zn1—C1—O2	82.18 (10)	C15—C16—C17—C18	175.64 (17)
N2—Zn1—C1—O1	−7.89 (11)	C21—C16—C17—C18	−2.4 (3)
N2—Zn1—C1—O2	172.35 (9)	C15—C16—C21—C20	−175.12 (16)
Zn1—O1—C1—O2	0.23 (14)	C17—C16—C21—C20	3.0 (3)
Zn1—O1—C1—C2	−178.65 (12)	C16—C17—C18—C19	0.1 (3)
Zn1—O2—C1—O1	−0.25 (15)	C20—C19—C18—C17	1.7 (3)
Zn1—O2—C1—C2	178.64 (11)	C22—C19—C18—C17	−177.64 (19)
Zn1—N2—C9—C10	178.82 (17)	C21—C20—C19—C18	−1.2 (3)
C13—N2—C9—C10	−0.8 (3)	C21—C20—C19—C22	178.15 (18)
Zn1—N2—C13—C12	179.33 (11)	C16—C21—C20—C19	−1.1 (3)

### Hydrogen-bond geometry (Å, º)

**Table:**

*D*—H···*A*	*D*—H	H···*A*	*D*···*A*	*D*—H···*A*
N3—H31···O2^i^	0.82 (2)	2.13 (3)	2.914 (2)	162 (2)
N3—H32···O7^i^	0.92 (3)	2.35 (2)	3.261 (2)	171 (2)
O4—H41···O7^ii^	0.75 (2)	2.04 (2)	2.7890 (17)	173 (3)
O4—H42···O8	0.76 (3)	1.89 (3)	2.6547 (18)	175 (3)
O5—H51···O7	0.80 (2)	1.83 (2)	2.6264 (17)	171 (3)
O5—H52···O1^iii^	0.74 (2)	2.05 (2)	2.7610 (17)	164 (2)
O6—H61···O3^iii^	0.75 (3)	2.05 (3)	2.7993 (19)	170 (3)
O6—H62···N1^iv^	0.76 (3)	2.17 (3)	2.918 (3)	170 (3)
C11—H11···O7^i^	0.93	2.49	3.415 (2)	177

Symmetry codes: (i) *x*−1, *y*+1, *z*; (ii) *x*−1, *y*, *z*; (iii) *x*+1, *y*, *z*; (iv) −*x*+1, −*y*, −*z*.
